# Resistance to classical scrapie in experimentally challenged goats carrying mutation K222 of the prion protein gene

**DOI:** 10.1186/1297-9716-43-8

**Published:** 2012-02-01

**Authors:** Pier Luigi Acutis, Francesca Martucci, Antonio D'Angelo, Simone Peletto, Silvia Colussi, Cristiana Maurella, Chiara Porcario, Barbara Iulini, Maria Mazza, Luana Dell'Atti, Fabio Zuccon, Cristiano Corona, Nicola Martinelli, Cristina Casalone, Maria Caramelli, Guerino Lombardi

**Affiliations:** 1 CEA, Italian Reference Laboratory for TSEs, Neuroscienze - Istituto Zooprofilattico Sperimentale del Piemonte, Liguria e Valle d'Aosta, Via Bologna 148, 10154 Torino, Italy; 2 Dipartimento di Patologia Animale - Facoltà di Medicina Veterinaria dell'Università degli studi di Torino, Via Leonardo Da Vinci 44, 10095 Grugliasco (TO), Italy; 3 Reparto Animali da laboratorio - Istituto Zooprofilattico Sperimentale della Lombardia e dell'Emilia Romagna, Via Bianchi 7/9 - 25124 Brescia, Italy

## Abstract

Susceptibility of sheep to scrapie, a transmissible spongiform encephalopathy of small ruminants, is strongly influenced by polymorphisms of the prion protein gene (*PRNP*). Breeding programs have been implemented to increase scrapie resistance in sheep populations; though desirable, a similar approach has not yet been applied in goats. European studies have now suggested that several polymorphisms can modulate scrapie susceptibility in goats: in particular, *PRNP *variant K222 has been associated with resistance in case-control studies in Italy, France and Greece. In this study we investigated the resistance conferred by this variant using a natural Italian goat scrapie isolate to intracerebrally challenge five goats carrying genotype Q/Q 222 (wild type) and five goats carrying genotype Q/K 222. By the end of the study, all five Q/Q 222 goats had died of scrapie after a mean incubation period of 19 months; one of the five Q/K 222 goats died after 24 months, while the other four were alive and apparently healthy up to the end of the study at 4.5 years post-challenge. All five of these animals were found to be scrapie negative. Statistical analysis showed that the probability of survival of the Q/K 222 goats versus the Q/Q 222 goats was significantly higher (*p *= 0.002). Our study shows that *PRNP *gene mutation K222 is strongly associated with resistance to classical scrapie also in experimental conditions, making it a potentially positive target for selection in the frame of breeding programs for resistance to classical scrapie in goats.

## Introduction

Scrapie is a naturally occurring transmissible spongiform encephalopathy (TSE) of sheep and goats, characterized by the accumulation in the central nervous system (CNS) of an abnormal isoform (PrP^Sc^) of a host-encoded cellular prion protein (PrP^C^) [1]. Natural scrapie in goats was first reported in France [2], followed by other cases worldwide: a state of the art review on goat scrapie in the European Union, including its epidemiology, was published by Vaccari et al. [3]. Goats are susceptible not only to classical scrapie, but also atypical/Nor98 scrapie cases have been detected. As in sheep, it is characterized by distinctive features in epidemiology (i.e., occurrence as single cases in the affected herds), molecular pattern, and distribution of histopathological changes [3]. Moreover, the only two confirmed cases of natural bovine spongiform encephalopathy (BSE) in small ruminants were reported in goats in France [4] and in a retrospective study in the UK [5].

The first Italian case of scrapie in the goat was diagnosed in 1997 [6]; since then, 65 outbreaks have occurred (51 of classical scrapie and 14 of Nor98 scrapie), in which 13 000 goats were culled in accordance with the foreseen measures for eradication of the disease.

Although an infectious disease, the susceptibility of sheep to scrapie is strongly influenced by polymorphisms of the prion protein gene (*PRNP*). *PRNP *haplotypes valine/arginine/glutamine (VRQ) and alanine/arginine/glutamine (ARQ) at codons 136, 154, 171, respectively, are associated with high susceptibility to classical scrapie, whereas the ARR haplotype has been linked to resistance [7-10]. Accordingly, the EU has implemented breeding programs to increase scrapie resistance in sheep populations. In compliance with regulation (EC) 999/2001, as amended, several Member States are now increasing the frequency of the ARR haplotype. A similar approach has not yet been applied in goats, but it would be desirable in this species too, given that scrapie poses a problem for the economy and animal welfare, and that goats, often bred in mixed flocks with sheep, can play a role in maintaining the circulation of scrapie strains and the consequent sheep exposure. Genetic analysis of the goat *PRNP *gene revealed 46 polymorphisms in the open reading frame [3,11], including silent mutations and a *PRNP *variant containing three instead of the five usual octapeptide repeats [12]. Various European studies have suggested that several polymorphisms can modulate scrapie susceptibility in goats as well. The presence of methionine (M) at codon 142 is associated with increased incubation periods after experimental challenge of BSE and scrapie strains [13] and in natural scrapie outbreaks [14,15]. A reduced susceptibility to natural scrapie has also been reported for goats carrying arginine (R) at codon 143, histidine (H) at codon 154 [16,17] or glutamine (Q) at codon 211 [14]. However, H154 has clearly been suggested to be a risk factor for Nor98 goat scrapie [18]. The most promising results have been obtained for codon 146, carrying serine (S) or aspartic acid (D), which is linked to high resistance in Cyprus [19,20], and for codon 222, carrying lysine (K), which in Italy was first reported as conferring resistance and has only been associated with healthy animals [16,21]. An association of K222 with a protective effect was also later found in France and Greece [14,22]. Taken together, these results provide encouraging evidence for the support of breeding programs for resistance in goats against classical scrapie in all EU Member States, as stated by the EFSA Panel on Biological Hazards in the "Opinion on genetic TSE resistance in goats in all EU Member States" [23], and perhaps also in other non-EU countries. In such a prospective, it is essential to reinforce existing data from field studies with those from experimental studies, particularly those carried out with the experimental transmission of different TSE isolates in goats harbouring the *PRNP *alleles of interest.

In this study, we investigated the resistance given by mutation K222 by intracerebrally challenging two groups of goats, with and without the considered allele, using a natural Italian goat scrapie isolate. Our study confirms the highly protective effect of K222 against classical scrapie. Furthermore, being the first scrapie transmission study carried out in goats in Italy, it provides information on clinical signs and PrP^Sc ^distribution patterns in goats affected by an Italian scrapie strain.

## Materials and methods

### Animals and animal care

Ten four-month-old goats were purchased from two herds with no record of scrapie cases in the last five years: six animals were Camosciata cross-bred and four were dwarf goat cross-bred. The animals were chosen on the basis of their genotype and having been born in northern Italy near the study site. This was done to avoid problems of stress due to long transport or climatic changes. The animals were previously genotyped at the *PRNP *locus, as described below, and two groups were formed: one group consisted of five animals heterozygous for Q/K at codon 222, and one group of five wild type Q/Q 222 animals, with a mixed assortment of the genotype at codon 240 (Table 1). No K/K 222 goats were found. In both groups there were three Camosciata cross-bred and two dwarf goat cross-bred animals. No other mutations were present in the *PRNP *gene of these animals, except for the two Q/Q 222 dwarf goat cross-bred animals that presented a deletion of two octarepeats, thus being 3/5 heterozygous for the octapeptide repeats. These animals were included in the study to also gather information on the possible resistance conferred by this deletion, as hypothesized by Goldmann et al. [12]. The animals were kept in the facility for one month before challenging to allow them to adapt to the new environment and to exclude the presence of other diseases. During the experiment, they were all kept in the same room to expose them to identical environmental conditions. All procedures involving the animals and their care were conducted in conformity with national and international laws and policies (EEC Council Directive 86/609, OJL358, 1, 12 December 1987; Italian Legislative Decree 116/92, Gazzetta Ufficiale della Repubblica italiana 10, 18 February 1992; and Guide for the Care and Use of Laboratory Animals, U.S. National Research Council, 1996).

**Table 1:** Goats included in the study: survival time and duration of symptoms after i.c. challenge.

*Ear tag*	*Sex*	*Breed*	*Genotype*	*Scrapie positivity*	*Survival time (days)*	*Symptomathology duration (days)*
1	F	Camosciata cross-bred	Q/Q222 P/P240	yes	553	5
12	F	Dwarf goat cross-bred	3/5 repeats Q/Q222 S/P240	yes	539	23
14	M	Dwarf goat cross-bred	3/5 repeats Q/Q222 S/P240	yes	539	13
18	F	Camosciata cross-bred	Q/Q222 S/P240	yes	477	14
VA2	F	Camosciata cross-bred	Q/Q222 S/S240	yes	734	2

					Mean value: 569 (± 97)	

7	M	Dwarf goat cross-bred	Q/K222 S/P240	no	1643	/
14R	F	Camosciata cross-bred	Q/K222 S/S240	no	720	/
17	F	Dwarf goat cross-bred	Q/K222 S/S240	no	1643	/
29	F	Camosciata cross-bred	Q/K222 S/P240	no	1643	/
32	F	Camosciata cross-bred	Q/K222 S/P240	no	1643	/

#### Genetic analysis

Genomic DNA was isolated from EDTA-treated blood samples using Thermo Labsystems KingFisher kits (Thermo LabSystems Inc., Beverly, MA, USA). PCR amplification of the entire open reading frame of the *PRNP *gene was performed according to a previously described protocol [24] using the primers p8(+) (5'-CAGGTTAACGATGGTGAAAAGCCACATAGG-3') and p9(-) (5'-GGAATTCTATCCTACTATGAGAAAAATGAGG-3') [8]. *PRNP *polymorphisms were detected by direct DNA sequencing on both strands of the PCR products by using dye terminator cycle sequencing and an ABI Prism 3130 Genetic Analyser (Applied Biosystems, Carlsbad, CA, USA). Sequencing primers were p8(+), p61(+) (5'-AACCAACATGAAGCATGTGG-3'), p60(-) (5'-GATAGTAACGGTCCTCATAG-3') and p9(-)[7]. The primers were hybridized to the target *PRNP *DNA at codons 1-7, 109-116, 147-154 and 249-257, respectively, (ovine reference sequence GenBank accession number AJ000739).

### Inoculum preparation and inoculation

The inoculum was prepared from the brain tissue of a female goat affected by natural classical scrapie (according to the approved discriminatory immunoblot carried out by the Istituto Superiore di Sanità, Rome, Italy) with genotype of the *PRNP *gene heterozygous at codon 240 (S/P) and with no other mutations. The inoculum was ground in a mechanical grinder and homogenized with normal sterile saline solution to a final concentration of 10% (w/v). A solution of penicillin and streptomycin was added to the inoculum prior to use and the homogenate was checked for microbiological sterility. Just prior to inoculation, the animals were clinically examined to rule out clinical abnormalities. Intracerebral inoculation of the kids was performed as previously described [25] with minor modifications. Briefly, the animals were anaesthetized with xylazine (50 μg/kg) and, in surgical aseptic conditions, a midline incision was made in the skin at the junction of the parietal and frontal bones and a 1 mm hole was drilled through the calvarium. The inoculum (0.5 mL of 10% w/v brain homogenate) was injected into the midbrain through a 22 gauge, 9 cm long needle while withdrawing the needle from the brain. The skin incision was closed with a single suture. The inoculated animals were housed together in a bio-safety level 3 containment facility.

### Clinical evaluation

Clinical evaluation comprised daily observation and biweekly physical assessment carried out by the animal husbandry staff and the veterinarian, respectively. Neurologic examination was performed monthly by a board-certified neurologist. For this purpose, a clinical examination protocol, previously used for sheep [26], was applied. The protocol followed the standard procedure for assessing mental status, posture, gait, postural reactions and proprioception, cranial nerves, spinal reflexes and sensitivity. Sensitivity to external stimuli was evaluated by light and acoustic response, as described for BSE [27], wherein an animal was considered hyperreactive if it showed an exaggerated response three times in a row. The nibble reflex was defined as positive if the animal showed head and neck extension, chewing movements associated with head and tongue movements after being manually stimulated on the withers and lumbosacral areas.

An animal was considered symptomatic (onset of symptoms) if it showed at least two of the following criteria: abnormal fleece, abnormal mental status/behaviour, abnormal gait, abnormal postural reaction/propioception and positive nibble reflex. Neurologic examinations were intensified according to the onset of symptoms. Animals that became inappetant or tended toward recumbency were humanely euthanized.

### Tissue sample collection

After general anaesthesia with propofol (PropoVet^®^, Abbott, Illinois, USA) administered intravenously (i.v.), the animals were euthanized with i.v. injected Enbutramide/Mebezonium iodide/Tetracaine hydrochloride (Tanax^®^, Intervet Inc. Merck, Summit, NJ, USA). After culling of the animals, the whole brain, the entire spinal cord, and the lymph reticular system were collected and divided into two halves, one was frozen at -80°C for immunoblot analyses and the other was fixed in 4% buffered formalin for immunohistopathological examinations. A further panel of peripheral organs and tissues was collected and frozen at -80°C for immunoblot analyses (Table 2).

**Table 2:** Tissues collected at necropsy.

*Central nervous system*	*Lymph reticular tissues*	*Respiratory tract*	*Gastro-intestinal tract*	*Muscles*	*Miscellaneous*
Encephalon	Tonsil	Trachea	Salivary glands	Tongue	Eye
Cerebellum	Retropharyngeal lymph node	Lung	Esophagus	Masseter muscle	Kidney
Brainstem	Mandibular lymph node	Olfactory tract at nasal septum, ethmoturbinates and ventral nasal concha	Liver	Biceps brachii muscle	Urinary bladder
Cervical spinal cord	Mesenteric lymph node		Rumen	Triceps femoris muscle	Mammary gland
Thoracic spinal cord	Spleen		Omasum	Heart	Adrenal gland
Lumbar spinal cord	Third eyelid lymphoid tissue		Reticulum		
			Abomasum		
			Duodenum		
			Jejunum		
			Ileum		
			Ileo-ciecal valve		
			Caecum		
			Rectum		

### Immunoblot analysis

#### Pre-treatment for extraneural tissues

Extraneural tissues, not including the spleen, were cut into small pieces by means of scalpels and incubated in a PBS-1% trypsin solution overnight at room temperature (RT) under gentle agitation. After incubation, the tissues were rinsed with ultrapure water [28]. From the authors' experience, trypsin digestion did not reduce the amount of detectable PrP^sc^.

#### PrP extraction method

Ten percent (w/v) homogenates from different brain areas and extraneural tissues were prepared in lysis buffer (10% N-lauroylsarcosine diluted in Tris Buffer Saline pH 7.4) and clarified by centrifugation at 22 000 *g *for 20 min at 10°C (Ultracentrifuge Optima TLX, Rotor TLA 55, Beckman Coulter, Fullerton, CA, USA). For extraneural tissues a further step was added in which supernatant was collected and incubated with benzonase nuclease 50 meq/mL (Novagen, San Diego, CA, USA) for 30 min at 37°C. The supernatants were incubated with proteinase K (PK; 40 μg/mL; Sigma Aldrich, Poole, Dorset, UK) for 1 h at 37°C under continuous shaking. After centrifugation at 215 000 *g *for 1 h at 10°C (Ultracentrifuge Optima TLX, Rotor TLA 110, Beckman Coulter), the pellet was dissolved in Laemmli buffer and boiled for 10 min at 99°C.

#### Electrophoresis and immunodetection method

Samples were subjected to sodium dodecyl sulphate polyacrilamide gel electrophoresis on a 12% handmade minigel (acrylamide/bisacrylamide ratio 37.5:1) and then transferred to a polyvinylidene fluoride membrane (PVDF) (Immobilion P, Millipore, Billerica, MA, USA) by wet blotting. PrP^Sc ^was detected by the P4 (0.1 μg/mL; R-Biopharm, Darmstadt, Germany) [29] monoclonal antibody and an anti-mouse antiserum conjugated with alkaline phosphatase. Reaction was revealed by a chemiluminescent substrate (Immun-Star, Bio-Rad, Hercules, CA, USA) and visualised on Hyperfilm ECL (GE-Healthcare Ltd., St. Giles, UK) or by a gel documentation analysis system (UVI Prochemi, Uvitec, Cambridge, UK). Comparison of the molecular masses of the PrP^Sc ^bands was carried out on the inoculated goats and inoculum sample. For quantitative study of the glycoform ratios, chemiluminescent signals corresponding to the three glycoforms of PrP^Sc ^were quantified using a UVI Prochemi analysis system. Glycoform ratios were expressed as mean percentages of the total signal for the three glycoforms (di-, mono-, and un-glycosylated) from three different runs of the samples.

### PrP^Sc ^quantification

In order to estimate the relative concentration of PrP^Sc ^in some positive extraneural organs (skeletal muscle, intestinal tract, omasum, abomasum, kidney), the signal intensity was quantified. Specifically, we compared the intensity of the proteinase-K digested positive signals with calibration curves obtained by diluting the corresponding brainstem of the scrapie-positive goat with scrapie-negative homogenates of the above-mentioned extraneural tissues. This comparison was done with three of the scrapie-positive animals. The extraction method and immunoblot technique were identical to those previously described. Quantification analyses were performed using the UVI Prochemi gel documentation and analysis system.

### Immunohistochemical analysis

Immunohistochemical investigations for PrP^Sc ^were performed on each brain area and lymph reticular tissue. Four-μm-thick sections of each formalin-fixed, paraffin-embedded specimen were cut. The slides were dewaxed and rehydrated by routine methods and then immersed in 98% formic acid for 25 min. After washing in distilled water, the sections were autoclaved for 30 min at 121°C in citrate buffer (pH 6.1). Endogenous peroxidase activity was blocked in 3% hydrogen peroxide for 20 min at RT. To block non-specific tissue antigens, the sections were incubated with 5% horse blocking serum for 20 min at RT and then incubated for 1 h at RT with primary monoclonal antibody L42 (epitope GNDYEDRYYRENMYRYPNQ, amino acids 145 to 163 of the ovine PrP, 1: 250 dilution; RIDA, R-Biopharm, Darmstadt, Germany) [30]. After rinsing, a biotinylated goat anti-mouse secondary antibody (1: 200 dilution; Vector Laboratories, Burlingame, CA, USA) was applied to the tissue sections for 30 min at RT, followed by the avidin-biotinperoxidase complex (Vectastain ABC kit; Vector Laboratories), according to the manufacturer's protocol. PrP^Sc ^immunoreactivity was visualized using 3,3'-diaminobenzidine (Dakocytomation, Carpinteria, CA) as a chromogen; the sections were then counterstained with Meyer's haematoxylin. In case of a positive result, specificity was checked by replacing the primary antibody by normal serum.

### Statistical analysis

Survival times between the two groups were compared using Kaplan-Meier survival estimates. The difference in the probability of survival was analyzed using the log-rank test for equality of survivor functions.

## Results

By the end of the study, all the susceptible Q/Q 222 goats had died or been euthanized for animal welfare after a mean incubation period of 19 months (569 ± 97 days). Survival times are shown in Table 1. All were shown to be scrapie positive by both immunoblot and immunohistochemistry. In the group of supposedly resistant Q/K 222 goats, one animal died of unknown cause 24 months into the study. All the other animals were alive and apparently healthy up to the end of the study, when they were euthanized, i.e., at 4.5 years post-challenge (Table 1). All five animals, including the one dead at 24 months post-challenge, were shown to be scrapie negative by immunoblot and immunohistochemistry of both the CNS and the lymph reticular system. Statistical analysis shows that the probability of survival of the Q/K 222 goats versus the Q/Q 222 goats was significantly higher (χ^2^= 9.34, *p *= 0.002).

At clinical examination, three animals (ear tag 18, 1 and VA2) showed itching, skin lesions, depressed mental status, wide base stance, tremors, ataxia, weakness and abnormal postural reactions/proprioception. Two animals (ear tag 12 and 14) developed, in addition to skin lesions, depression and tremors, a central vestibular syndrome manifested by head tilt, wide base stance, asymmetrical ataxia and circling, ipsilateral paresis and proprioceptive deficits, vertical nystagmus, positional strabismus, bilaterally decreased menace reaction and normal palpebral reflex. Table 1 reports the duration of the symptoms.

All five Q/Q 222 animals tested positive for the CNS and the large part of the lymph reticular system analysed both by Western blot and immunohistochemistry. Moreover, the peripheral nerves and numerous extraneural organs tested scrapie positive by Western blot (Table 3). Molecular analysis of PrP^Sc ^of the inoculated goats and the inoculum sample revealed a similar electrophoretic pattern characterized by three bands corresponding to the di-, mono- and un-glycosylated forms, with a molecular weight of 30, 25 and 20 kDa, respectively. The mean (± standard deviation [SD]) di-, mono- and un-glycosylated band intensity was 48.16 ± 2: 31.86 ± 2: 19.98 ± 1.9, respectively, for the inoculated goats and 49.27 ± 1.5: 32.19 ± 1.1: 18.54 ± 2, respectively, for the inoculum sample. Quantification showed that the PrP^Sc ^signal intensity in the positive muscular tissues was generally similar to that obtained when five milligrams of the corresponding positive brainstem homogenate were diluted in five grams of a negative muscle homogenate. From this finding, we estimated that the PrP^Sc ^levels in the three examined cases were lower than those found in the corresponding brainstems by a factor of approximately 1 × 10^-3^. The signal intensities of the positive omasum, abomasum and kidney were usually lower than those found in the corresponding brainstems by a factor of 1 × 10^-3 ^- 1 × 10^-4^.

**Table 3:** Results of Wb and IHC analyses of the Q/Q 222 goats.

Apparatus		Central Nervous System																										Peripheral Nervous System		
Organs/Tissues		Brainstem		Cerebellum		Occipital cortex		Parietal cortex		Temporal cortex		Frontal cortex		Pyriform lobus		Mesence - phalon		Talamus		Basal nuclei		Cervical spinal cord		Thoracic spinal cord		Lumbar spinal cord		Optic nerve	Brachial nerve	Sciatic nerve
**ID sample**	**#1**	**+**	**+**	**+**	**+**	**+**	**+**	**+**	**+**	**+**	**+**	**+**	**+**	**+**	**+**	**+**	**+**	**+**	**+**	**+**	**+**	**+**	**+**	**+**	**+**	**+**	**+**	+	+	+
	**#12**	**+**	**+**	**+**	**+**	**+**	**+**	**+**	**+**	**+**	**+**	**+**	**+**	**+**	**+**	**+**	**+**	**+**	**+**	**+**	**+**	**+**	**+**	**+**	**+**	**+**	**+**	+	+	+
	**#14**	**+**	**+**	**+**	**+**	**+**	**+**	**+**	**+**	**+**	**+**	**+**	**+**	**+**	**+**	**+**	**+**	**+**	**+**	**+**	**+**	**+**	**+**	**+**	**+**	**+**	**+**	+	+	+
	**#18**	**+**	**+**	**+**	**+**	**+**	**+**	**+**	**+**	**+**	**+**	**+**	**+**	**+**	**+**	**+**	**+**	**+**	**+**	**+**	**+**	**+**	**+**	**+**	**+**	**+**	**+**	+	+	+
	**#VA2**	**+**	**+**	**+**	**+**	**+**	**+**	**+**	**+**	**+**	**+**	**+**	**+**	**+**	**+**	**+**	**+**	**+**	**+**	**+**	**+**	**+**	**+**	**+**	**+**	**+**	**+**	+	+	+
***positive/*** ***tested***		5/5	5/5	5/5	5/5	5/5	5/5	5/5	5/5	5/5	5/5	5/5	5/5	5/5	5/5	5/5	5/5	5/5	5/5	5/5	5/5	5/5	5/5	5/5	5/5	5/5	5/5	5/5	5/5	5/5
***method***		*Wb*	*IHC*	*Wb*	*IHC*	*Wb*	*IHC*	*Wb*	*IHC*	*Wb*	*IHC*	*Wb*	*IHC*	*Wb*	*IHC*	*Wb*	*IHC*	*Wb*	*IHC*	*Wb*	*IHC*	*Wb*	*IHC*	*Wb*	*IHC*	*Wb*	*IHC*	*Wb*	*Wb*	*Wb*

**Apparatus**		**Lymph reticular System**														Respiratory tract						**Digestive apparatus**								
**Organs/Tissues**		**Tonsil**		**Mandibular lymph node**		**Retro-** **pharingeal** **lymph node**		**Mesenteric lymph node**		**Spleen**		**Ileo-ciecal valve**		**Third** **eyelid**		Olfactory tract		Trachea		Lung		**Salivary glands**		**Esophagus**		**Liver**		**Rumen**	**Omasum**	**Reticulum**
**ID sample**	**#1**	**+**	**+**	**+**	**+**	**+**	**+**	**+**	**+**	**+**	**+**	**+**	**+**	**+**	**+**	+		-		-		**+**		**+**		**-**		**-**	**+**	**+**
	**#12**	**+**	**+**	**+**	**+**	**+**	**+**	**+**	**+**	**+**	**+**	**+**	**-**	**-**	**-**	+		-		-		**-**		**+**		**-**		**-**	**+**	**+**
	**#14**	**+**	**+**	**+**	**+**	**+**	**-**	**+**	**+**	**+**	**+**	**+**	**+**	**-**	**-**	+		-		-		**-**		**-**		**-**		**-**	**+**	**+**
	**#18**	**+**	**+**	**+**	**+**	**+**	**+**	**+**	**+**	**+**	**+**	**+**	**+**	**+**	**+**	+		-		-		**-**		**-**		**-**		**-**	**-**	**-**
	**#VA2**	**+**	**+**	**+**	**+**	**+**	**+**	**+**	**+**	**+**	**+**	**+**	**+**	**-**	**-**	-		-		-		**-**		**-**		**-**		**-**	**-**	**-**
***positive/*** ***tested***		*5/5*	*5/5*	*5/5*	*5/5*	*5/5*	*4/5*	*5/5*	*5/5*	*5/5*	*5/5*	*5/5*	*4/5*	*2/5*	*2/5*	*4/5*		*0/5*		*0/5*		*1/5*		*2/5*		*0/5*		*0/5*	*3/5*	*3/5*
***method***		*Wb*	*IHC*	*Wb*	*IHC*	*Wb*	*IHC*	*Wb*	*IHC*	*Wb*	*IHC*	*Wb*	*IHC*	*Wb*	*IHC*	*Wb*		*Wb*		*Wb*		*Wb*		*Wb*		*Wb*		*Wb*	*Wb*	*Wb*

**Apparatus**		Gastrointestinal tract												**Muscles**										Miscellaneous						
**Organs/Tissues**		Abomasum		Duodenum		Jejunum		Ileum		Caecum		Rectum		**Tongue**		**Masseter muscle**		**Biceps brachii muscle**		**Triceps femoris muscle**		**Heart**		Eye		Kidney		Urinary bladder	Mammary gland	Adrenal gland
**ID sample**	**#1**	-		+		+		+		+		+		**-**		**+**		**+**		**+**		**-**		+		+		+	-	-
	**#12**	-		+		+		+		+		+		**+**		**+**		**+**		**+**		**+**		+		-		-	-	-
	**#14**	-		+		+		+		+		+		**+**		**-**		**-**		**+**		**+**		+		-		-	/	-
	**#18**	-		-		-		-		-		-		**-**		**-**		**-**		**-**		**-**		-		-		-	-	-
	**#VA2**	-		+		+		+		+		-		**+**		**-**		**-**		**-**		**-**		-		-		-	-	-
***positive/*** ***tested***		*0/5*		*4/5*		*4/5*		*4/5*		*4/5*		*3/5*		*3/5*		*2/5*		*2/5*		*3/5*		*2/5*		*3/5*		*1/5*		*1/5*	*0/4*	*0/5*
***method***		*Wb*		*Wb*		*Wb*		*Wb*		*Wb*		*Wb*		*Wb*		*Wb*		*Wb*		*Wb*		*Wb*		*Wb*		*Wb*		*Wb*	*Wb*	*Wb*

+: positive result; -: negative result

The Q/K 222 goats were completely negative by immunoblot analysis of all extraneural tissues and organs.

## Discussion

Our study shows that *PRNP *gene mutation K222 is strongly associated with resistance to classical scrapie in experimentally challenged goats, confirming the results previously obtained in field studies [16,21]. The Q/K 222 goats arrived as healthy, scrapie negative until the end of the study, with a significantly longer survival time of about three years compared to the Q/Q 222 inoculated goats. The study had to be necessarily concluded, but the survival time of 1643 days (i.e. 4.5 years), which is nearly equal to the average economic lifespan of a goat in Italy (i.e. five years), was long enough to allow the hypothesis for a high level of resistance, even if a minimal susceptibility or a very prolonged incubation period (i.p.) cannot be excluded. Admittedly, the transmission of infection was carried out in conditions where the host barriers were reduced to a minimum, via the intracerebral route and by inoculating with a goat natural scrapie isolate that was thus already adapted to the species. Nonetheless, the Q/K 222 goats remained healthy for a longer period than the i.p. reported in other transmission studies in goats and sheep. Heterozygous goats with the partial resistance-associated M142 mutation succumbed much earlier, at 984-985, 675-894 and 640-895 days, after i.c. transmission of BSE, sheep scrapie CH1641 and sheep-passaged ME7, respectively. In wild type goats, instead, the same strains had an i.p. similar to that obtained in the present study [13]. Similarly, Foster et al. [31] reported in wild type goats i.c. challenged with natural scrapie and BSE, an i.p. of 362-517 and 506-570 days, respectively. In sheep, i.c. BSE challenged ARR/ARR animals showed an i.p. of 1008-1127 days post-infection [32]. However, because our study showed the resistant goats still healthy, it cannot be excluded that they might have remained scrapie negative for their entire normal life span.

The results of the challenge of the Q/Q 222 goats yielded further interesting information on goat scrapie genetics. The two goats carrying the octarepeat deletion died of scrapie, with an i.p. not unlike that of the other goats in the same group, showing that in our sample this mutation did not confer any resistance, on the contrary to that which was hypothesized by Goldmann [12], perhaps because of the scrapie strain used in the experiment. It is noteworthy that one Q/Q 222 goat (VA2) died after an i.p. nearly 200 days longer than that of the rest of the same group. This goat had no other mutation on the *PRNP *gene, but it was the only one out of its group that was homozygote for serine at codon 240. While this prolonged i.p. in one animal does not allow us to say whether it was significant or just happened by chance, we can speculate that other unknown genetic factors may have come into play or that indeed serine at codon 240 could have had an effect. Field studies have produced controversial results about codon 240, indicating a positive association with scrapie infection of P240 [21] or a positive association with clinical disease of S240 [22] or no association [13,16,17]. Interestingly, Goldmann et al. [13] reported that a BSE orally challenged S/S 240 goat had an i.p. 500 days longer than an S/P240 goat. Barillet et al. [14] found that mutation M142 in heterozygotes conferred a protective effect only when in association with P/P 240 homozygotes. Although codon 240 is generally not believed to influence scrapie susceptibility, it could exert an effect, perhaps depending on the scrapie strain involved.

The clinical signs seen in three of the five scrapie positive animals were similar to those previously described in both experimental and natural scrapie, but none of the animals in the present study showed aggressiveness, hypersensitivity to external stimuli or hyperexcitability. As already known in goats, unlike that observed in sheep with scrapie, [31,33-35], scratching of the animal's back did not elicit the nibble reflex in any goat. Worth noting was the lateralization of clinical signs in the two remaining animals (ear tag 12 and 14), suggesting an involvement of the vestibular system. Histopathological examination of the brain ruled out a secondary disease that could have caused the asymmetrical clinical presentation. A vestibular syndrome in two goats with classical scrapie was described by Konold et al. [34]. This atypical clinical presentation stresses the importance of not excluding scrapie on the basis of lateralization of neurological signs in experimental and field conditions.

Since the route of inoculation differed from that of the natural disease, no conclusions on the pathogenesis of scrapie in goats can be drawn from the pattern of PrP^Sc ^distribution in the extraneural tissues; nonetheless, it does give information on the centrifugal diffusion of PrP^Sc^, which may prove useful for risk assessment of human exposure given by peripheral districts. Our results are in line with previous observations in goats and sheep with natural scrapie or after oral challenge. It appears that PrP^Sc ^had a widespread distribution, similar to that observed in sheep, involving the lymph reticular system, as already known in goats [36], and other districts, some of which have never been reported in goats so far, such as the omasum, abomasum, kidney and the olfactory system, have already been reported to be sites of PrP^Sc ^deposition in sheep [37,38,28]. Similarly to what has been found recently in naturally affected sheep and goats [39], scrapie positivity was not found in the third eyelid or the rectal mucosa in all the animals, highlighting the limitation of using those districts for preclinical diagnosis, as recently proposed [40,41].

The finding of positive signals in the skeletal muscles in three of the five scrapie-positive goats is consistent with reports of muscle tissues that tested positive in a scrapie i.c. challenged goat [42] and orally challenged goats older than 21 months [43]. The PrP^Sc ^amount, compared to that in the brainstem, was at least 1000-fold lower in the muscles and 1000 to 10 000-fold lower in the omasum, abomasum and kidney, suggesting a low risk for human transmission by these tissues.

After identifying a genetic variant associated with TSE resistance, the main question is the validity of the association in relation to different classical scrapie strains, atypical scrapie and BSE. Our study confirms that variant K222 of the goat *PRNP *gene can be a genetic target to select for in the frame of breeding programs for the control and eradication of classical scrapie in goats. This result is certainly mainly valid for Italy, given that an Italian scrapie isolate was used in the experiment. The situation in Italy could be particularly favorable because there is good evidence for a low variability of circulating scrapie strains [44,45]. The association of K222 with resistance found in field studies in France and Greece indicates that this variant can probably confer resistance to several classical scrapie strains. Moreover, a recent study showed that cell-free conversion of recombinant PrP was completely abolished in the presence of the recombinant K222 variant, using mouse scrapie strain ME7 [46]. On the contrary, some positive Q/K 222 goats were recently detected in Greece, with some evidence that they could be affected by a scrapie strain different from those previously studied in Italy, France and Greece, from which K222 carriers could not have been protected [11]. No data are currently available regarding the K222 variant and BSE and atypical scrapie. In Italy, a Nor98 positive goat carrying K222 in linkage with H154 was found, suggesting that K222 might not confer resistance to atypical scrapie [18]. Similarly, in a highly scrapie-affected Greek herd, a unique C- and N-terminally truncated protease resistant PrP fragment, which has been suggested to be a marker of an unrecognized prion protein disorder, was identified in clinically healthy goats, including Q/K 222 animals as well [47].

One limitation of the present study was the lack of homozygous K/K222 animals. The role of homozygotes needs to be investigated to verify whether there could be a situation like that in sheep, where ARR/ARR animals are highly resistant, while some cases of disease are occasionally reported in ARR/XXX [48]. Barillet et al. [14] estimated the risk for Q/K 222 goats as being within a similar range as the risk for ARR/ARQ sheep, comparing them to their respective wild type genotypes. Moreover, the study on cell-free conversion showed that recombinant K222 alone abolished conversion and significantly reduced conversion in a condition of simulated heterozygosis, i.e., in co-incubation with another PrP variant [46]. These results thus suggest that K/K 222 homozygotes should be more resistant than heterozygotes against classical scrapie, even if only experimental challenges can exclude the possible phenomenon of overdominance, as observed in the susceptibility of mice to several scrapie strains [49].

Another open question in the present study is whether negative K222 goats can be healthy carriers of infection. To answer this, the negative tissues of the challenged Q/K 222 goats will be tested by bioassay for the presence of infectivity.

Other experimental challenge studies on the K222 variant are underway in Europe [23]. If they obtain the same results as ours in other breeds and with other strains including BSE, it will be possible to implement breeding programs to control classical scrapie in goats in the near future.

## Competing interests

The authors declare that they have no competing interests.

## Authors' contributions

PLA conceived and led the project, interpreted the genetics and drafted the manuscript; FM prepared the inoculum, assisted in sample collection, performed PrP^Sc ^quantification, and contributed to the manuscript; ADA carried out the neurological clinical evaluations, interpreted the clinics, and contributed to the manuscript; SP assisted in observation of the animals, sample collection, and interpreting the results of genetics; SC carried out the genetic analysis to find the animals and kept the database and records; CM performed the statistical analysis and contributed to the manuscript; CP carried out the immunohistochemistry; BI assisted in sample collection and interpreting the immunohistochemistry results; MM interpreted the immunoblot results; LDA carried out the immunoblot analysis; FZ assisted in observation of the animals; CC prepared the inoculum and assisted in sample collection; NM managed daily observation of the animals and assisted in sample collection; CC interpreted the immunohistochemistry results; MC contributed to the manuscript; GL inoculated the animals and was responsible for their care. All the authors read and approved the final manuscript.
